# Longitudinal BMI change and outcomes in Chronic Obstructive Pulmonary Disease: a nationwide population-based cohort study

**DOI:** 10.1186/s12931-024-02788-0

**Published:** 2024-03-30

**Authors:** Taeyun Kim, Sun Hye Shin, Hyunsoo Kim, Yunjoo Im, Juhee Cho, Danbee Kang, Hye Yun Park

**Affiliations:** 1 Division of Pulmonary and Critical Care Medicine, Department of Internal Medicine, Kosin University Gospel Hospital, Kosin University College of Medicine, Busan, Republic of Korea; 2 Division of Pulmonary and Critical Care Medicine, Department of Internal Medicine, Samsung Medical Center, Sungkyunkwan University School of Medicine, 81 Irwon-ro, Seoul, 06351 Republic of Korea; 3 Center for Clinical Epidemiology, Samsung Medical Center, Seoul, Republic of Korea; 4 Department of Clinical Research Design and Evaluation, SAIHST, Sungkyunkwan University, 115 Irwon-ro, Seoul, 06335 South Korea

**Keywords:** COPD, BMI, Mortality, Exacerbation, K-NHIS

## Abstract

**Background:**

The association between longitudinal body mass index (BMI) change and clinical outcomes in patients with chronic obstructive pulmonary disease (COPD) has not fully investigated.

**Methods:**

This retrospective cohort study included 116,463 COPD patients aged ≥ 40, with at least two health examinations, one within 2 years before and another within 3 years after COPD diagnosis (January 1, 2014, to December 31, 2019). Associations between BMI percentage change with all-cause mortality, primary endpoint, and initial severe exacerbation were assessed.

**Results:**

BMI decreased > 5% in 14,728 (12.6%), while maintained in 80,689 (69.2%), and increased > 5% in 21,046 (18.1%) after COPD diagnosis. Compared to maintenance group, adjusted hazard ratio (aHR) for all-cause mortality was 1.70 in BMI decrease group (95% CI:1.61, 1.79) and 1.13 in BMI increase group (95% CI:1.07, 1.20). In subgroup analysis, decrease in BMI showed a stronger effect on mortality as baseline BMI was lower, while an increase in BMI was related to an increase in mortality only in obese COPD patients with aHRs of 1.18 (95% CI: 1.03, 1.36). The aHRs for the risk of severe exacerbation (BMI decrease group and increase group vs. maintenance group) were 1.30 (95% CI:1.24, 1.35) and 1.12 (95% CI:1.07, 1.16), respectively.

**Conclusions:**

A decrease in BMI was associated with an increased risk of all-cause mortality in a dose-dependent manner in patients with COPD. This was most significant in underweight patients. Regular monitoring for weight loss might be an important component for COPD management.

**Supplementary Information:**

The online version contains supplementary material available at 10.1186/s12931-024-02788-0.

## Background

Chronic obstructive pulmonary disease (COPD) is characterized by persistent respiratory symptoms and airflow limitation [1]. However, it is a complex and heterogeneous disease with symptoms and pathophysiological features that vary among individuals despite a similar degree of airflow obstruction [1]. Physical features, especially body mass index (BMI), vary widely in patients with COPD, ranging from underweight to morbidly obese. Low BMI in patients with COPD is generally associated with poor outcomes, including increased mortality, exacerbation, and lung function decline [2–4]. However, data regarding health and obesity in patients with COPD are conflicting. Several studies have suggested that being overweight or obese protects against exacerbation and mortality in COPD [2–4], which is more apparent in patients with severe disease [5]. In contrast, other studies have shown an increase in mortality in obese COPD patients [6, 7]. However, these previous studies have evaluated the relationship between BMI and outcomes in patients with COPD based on BMI measured at one point, rather than considering changes in BMI [2–4, 6, 7].

The effects of weight change on COPD-related outcomes have also been reported. A study conducted several decades ago as part of the Copenhagen City Heart Study revealed a significant dose-dependent association between weight loss and all-cause mortality [8]. However, this study recruited participants several decades ago, when recent treatment strategies for COPD were not yet being applied. In previous studies that included Asians for the consideration of differences in comorbidities and BMI among race and ethnicity groups [9], studies in large Japanese and Korean cohorts found that weight or BMI reduction was associated with higher exacerbation and overall mortality [10, 11]. However, these studies included few patients whose BMI changed and weight change was assessed by questionnaire.

In this regard, this study aimed to evaluate the relationship between BMI changes and clinical outcomes in patients with COPD using a large nationally representative cohort from Korea by specifically investigating the association across different BMI groups based on the classification for Asians. This would enable clinicians to guide patients to make appropriate lifestyle modifications.

## Materials and methods

### Data source

We conducted a retrospective cohort study using data from the Korean National Health Insurance System (K-NHIS) database, which covers the entire South Korean population. This comprehensive database contains extensive information on demographics, medical treatments, procedures, prescription drugs, diagnostic codes, and hospital utilization. Diagnoses in the K-NHIS database were classified according to the International Classification of Diseases, 10th revision (ICD-10). Regular audits of the ICD-10 codes, procedure records, and prescription records are conducted by the K-NHIS to ensure accuracy and prevent unnecessary medical expenses. Moreover, the K-NHIS claims database incorporates data from the national health screening examination, a standardized health screening program provided to all insured individuals every two years [12]. Approximately 76% of the target population participated in the health screening examination [12]. The data collected during the health screening examination included a self-administered questionnaire on medical history, lifestyle habits, anthropometric measurements, and laboratory tests [12]. Health examination facilities are designated and regulated by the relevant national laws to ensure quality control. For more detailed information on the NHIS database and health examinations, please refer to previous publications [12, 13].

### Study population

Our database included all COPD patients aged ≥ 40 years between January 1, 2014, and December 31, 2019. COPD was defined as the presence of code J43-J44 (except J43.0) (ICD-10) and the prescription of COPD medication at least twice within a year. Medications for COPD include long-acting muscarinic antagonists (LAMAs), long-acting beta-2 agonists (LABAs), inhaled corticosteroids (ICS) plus LABAs, short-acting muscarinic antagonists, short-acting beta-2 agonists, methylxanthines, systemic beta-agonists, and phosphodiesterase-4 inhibitors [14–16].

As the purpose of this study was to evaluate BMI changes after COPD diagnosis in terms of mortality and severe exacerbation, we included patients who had health examination data within 2 years before (Exam 1) and within 3 years after (baseline, Exam 2) the date of COPD diagnosis. A period of 3 years was chosen *a priori* based on previous literature, as well as the anticipated sample size and follow-up duration [17, 18]. After excluding 10,126 participants who had cancer before the Exam 2, 118,849 participants remained. Furthermore, to minimize potential reverse causality, we excluded 2,386 participants who developed any cancer or died within the first 6 months of follow-up from the Exam 2 (index date). The final sample size was 116,463, and the median duration of follow-up from the index date was 3.9 years (interquartile range [IQR]: 2.5–5.1 and range: 0.5–7).

The Institutional Review Board of the Samsung Medical Center approved the study (approval no:2022-09-022) and waived the requirement for informed consent because the K-NHIS data were deidentified. The study was conducted in accordance with the principles of the Declaration of Helsinki.

### Measurement

During each health examination, weight and height were measured by trained nurses. BMI was calculated as weight in kilograms divided by height in meters squared and was classified according to Asian-specific criteria (underweight, BMI < 18.5 kg/m^2^; normal weight, BMI 18.5 to 22.9 kg/m^2^; overweight, BMI 23 to 24.9 kg/m^2^; and obese, BMI ≥ 25 kg/m^2^) [19, 20]. BMI change (%) was calculated as the difference in BMI from the last examination within 2 years before COPD diagnosis (Exam 1) to the last examination within 3 years after COPD diagnosis (baseline, Exam 2), then the BMI difference was divided by the BMI at Exam 1 and multiplied 100. Participants were classified into three categories: decrease in BMI > 5%, increase in BMI > 5%, and no change (not more than 5%) [21, 22].

The primary endpoint was all-cause mortality rate. Any death events were recorded after Exam 2. The vital status and cause of death were obtained from death certifications collected by Statistics Korea from the Ministry of Strategy and Finance of South Korea [23].

The secondary endpoint was initial severe exacerbation after Exam 2. Severe exacerbation of COPD was defined as an hospitalization or emergency room visit with one of the following ICD-10 codes as the principal or secondary diagnosis: COPD (J43.X [except J43.0] or J44.X) or COPD-related disease (pneumonia [J12.X–J17.X], pulmonary thromboembolism [I26, I26.0, or I26.9], dyspnea [R06.0], or acute respiratory distress syndrome [J80]), and a prescription for systemic steroids or antibiotics at the same visit [24]. To minimize reverse causality (i.e., previous severe exacerbation could both affect low BMI and subsequent exacerbation), patients without a history of previous severe exacerbations between COPD diagnosis and Exam 2 were included for the analysis of severe exacerbation (*N* = 108,067).

Data on covariates were collected during Exam 2. Study participants completed a self-administered questionnaire with questions on medical history and lifestyle habits, including smoking and alcohol use in Exam 2 and medication use (LABA, LAMA, or ICS) within 1 year before Exam 2.

Residential areas and income levels were obtained from insurance eligibility. The residential areas were categorized as metropolitan cities (Seoul, Busan, Daegu, Daejeon, Gwangju, Incheon, and Ulsan). Income levels were categorized as Medical Aid, ≤ 30th, 30–70th, or > 70th percentile.

Comorbidities during the year before Exam 2 were obtained from claims data defined using ICD-10 codes and summarized using the Charlson Comorbidity Index (CCI) [25]. In addition to CCI, pulmonary tuberculosis (ICD-10: A15, A16, B90.9), interstitial lung disease (ICD-10: J84), bronchiectasis (ICD-10: J47), and pneumonia (ICD-10: J11–J18, J69) were determined using insurance claims data during a 1-year look-back period from Exam 2.

### Statistical analysis

The incidence rates were calculated as the number of events per 100 person-years of follow-up. The cumulative incidence of each outcome was estimated using the Kaplan–Meier method, and log-rank tests were used to evaluate the differences between groups. We calculated the hazard ratio (HR) with a 95% confidence interval (CI) for all-cause mortality and severe exacerbations comparing participants with > 5% increase and > 5% decrease in BMI versus those who had maintained BMI during follow-up. The proportionality of hazards was confirmed by visual inspection of log-minus-log plots and Schoenfeld residuals. The models were adjusted for age, sex, smoking status, drinking status, residential area, income, CCI, regular moderate-to-vigorous physical activity (MVPA), previous severe exacerbation within a year before Exam 2 (baseline), medication use (ICS, LABA, or LAMA) within the year before Exam 2 (baseline), pulmonary tuberculosis, bronchiectasis, and pneumonia. The covariables were selected *a priori* based on their possible associations with BMI changes and outcomes.

In addition, we modeled BMI change as a continuous variable using restricted cubic splines with knots at the 5th, 35th, 65th, and 95th percentiles of the sample distribution to provide a flexible estimate of the dose-response relationship between BMI change and mortality incidence.

In subgroup analysis, we examined the association between percentage BMI change and mortality by BMI categories before COPD diagnosis (underweight, normal, overweight, or obese). A sensitivity analysis was additionally performed according to the BMI category based on the World Health Organization (WHO): underweight (< 18.5 kg/m^2^), normal (18.5–24.9 kg/m^2^), Overweight (25–29.9 kg/m^2^), and Obese (≥ 30 kg/m^2^).

All statistical analyses were performed using SAS version 9.4 (SAS Institute Inc., Cary, NC, USA) and R version 4.0.3 (R Foundation for Statistical Computing, Vienna, Austria).

## Results

Of the 116,463 patients with COPD (median age, 67 years; male, 66%), 14,728 (12.6%) experienced a > 5% decrease in BMI, 80,689 (69.2%) maintained their BMI, and 21,046 (18.1%) experienced a > 5% increase in BMI after COPD diagnosis (Table 1). With respect to baseline BMI, 31.5% of underweight patients had a > 5% increase in BMI and 14.1% of obese patients had a < 5% decrease in BMI (Supplementary Table 1). Compared to those with maintained BMI, individuals with decreased and increased BMI were more likely to experience severe exacerbations in the previous year (6.6% for maintained BMI vs. 9.8% for decreased BMI vs. 7.8% for increased BMI, *p* < 0.001). Co-existing pulmonary diseases (history of pulmonary tuberculosis, interstitial lung disease, and pneumonia) and comorbidities were more prevalent in the BMI decrease group compared to BMI maintenance group (*p* < 0.001, Table 1).

**Table 1:** Characteristics of participants according to BMI change

	Over 5% decrease	Maintenance	Over 5% increase	*p* value
	*N* = 14,728	*N* = 80,689	*N* = 21,046	
**Age, years**	69.5 (10.5)	67.2 (9.9)	67.4 (10.2)	< 0.001
**Sex**				< 0.001
Male	9,405 (63.9)	54,231 (67.2)	13,646 (64.8)	
Female	5,323 (36.1)	26,458 (32.8)	7,400 (35.2)	
**Residential area, metropolitan**	7,172 (48.7)	43,204 (53.5)	10,893 (51.8)	< 0.001
**Income**				< 0.001
Medical Aid	773 (5.2)	2,664 (3.3)	1,180 (5.6)	
≤ 30th	3,212 (21.8)	17,743 (22.0)	4,665 (22.2)	
31st – 70th	4,799 (32.6)	26,250 (32.5)	6,888 (32.7)	
> 70th	5,721 (38.8)	32,712 (40.5)	7,986 (37.9)	
Unknown	223 (1.5)	1,320 (1.6)	327 (1.6)	
**BMI before COPD diagnosis**	24.3 (3.7)	24.1 (3.3)	22.9 (3.4)	< 0.001
Underweight	677 (4.6)	3,122 (3.9)	1,746 (8.3)	
Normal	4,853 (33.0)	26,868 (33.3)	9,565 (45.4)	
Overweight	3,379 (22.9)	20,425 (25.3)	4,666 (22.2)	
Obese	5,819 (39.5)	30,274 (37.5)	5,069 (24.1)	
**BMI after COPD diagnosis**	22.2 (3.4)	24.2 (3.4)	24.9 (3.7)	< 0.001
Underweight	1,987 (13.5)	3,181 (3.9)	482 (2.3)	
Normal	7,031 (47.7)	26,473 (32.8)	5,916 (28.1)	
Overweight	2,939 (20.0)	20,246 (25.1)	4,997 (23.7)	
Obese	2,771 (18.8)	30,789 (38.2)	9,651 (45.9)	
**Smoking status**				< 0.001
Never	6,551 (44.5)	33,075 (41.0)	8,711 (41.4)	
Past	2,968 (20.2)	19,352 (24.0)	5,585 (26.5)	
Current	3,107 (21.1)	16,393 (20.3)	3,719 (17.7)	
Unknown	2,102 (14.3)	11,869 (14.7)	3,031 (14.4)	
**Drinking status**				< 0.001
No	10,338 (70.2)	50,087 (62.1)	13,570 (64.5)	
Yes	4,387 (29.8)	30,562 (37.9)	7,470 (35.5)	
Unknown	3 (0.0)	40 (0.0)	6 (0.0)	
**Regular MVPA, yes**	3,199 (21.7)	19,445 (24.1)	4,493 (21.3)	< 0.001
**Charlson Comorbidity Index** ^a)^	2.4 (1.9)	2.1 (1.7)	2.2 (1.7)	< 0.001
**Comorbidities** ^a)^				
Pulmonary TB	613 (4.2)	2,508 (3.1)	834 (4.0)	< 0.001
ILD	409 (2.8)	1,589 (2.0)	385 (1.8)	< 0.001
Bronchiectasis	950 (6.5)	4,978 (6.2)	1,256 (6.0)	0.175
Pneumonia	3,081 (20.9)	14,066 (17.4)	3,835 (18.2)	< 0.001
**Medication** ^a)^				
LABA	2,027 (13.8)	11,228 (13.9)	3,058 (14.5)	0.048
LAMA	2,619 (17.8)	13,333 (16.5)	3,806 (18.1)	< 0.001
ICS	3,059 (20.8)	14,545 (18.0)	4,003 (19.0)	< 0.001
**Previous severe exacerbation** ^a)^	1,450 (9.8)	5,311 (6.6)	1,635 (7.8)	< 0.001

Values were presented as n (%) or mean (SD)

^a)^ Values were assessed within 1 year before Exam 2 (baseline)

BMI, body mass index; MVPA, moderate-to-vigorous physical activity; ICS, inhaled corticosteroid; LABA, long-acting beta-2 agonist; LAMA, long-acting muscarinic agonist; TB, tuberculosis; ILD, interstitial lung disease; SD, standard deviation

**Fig. 1:**
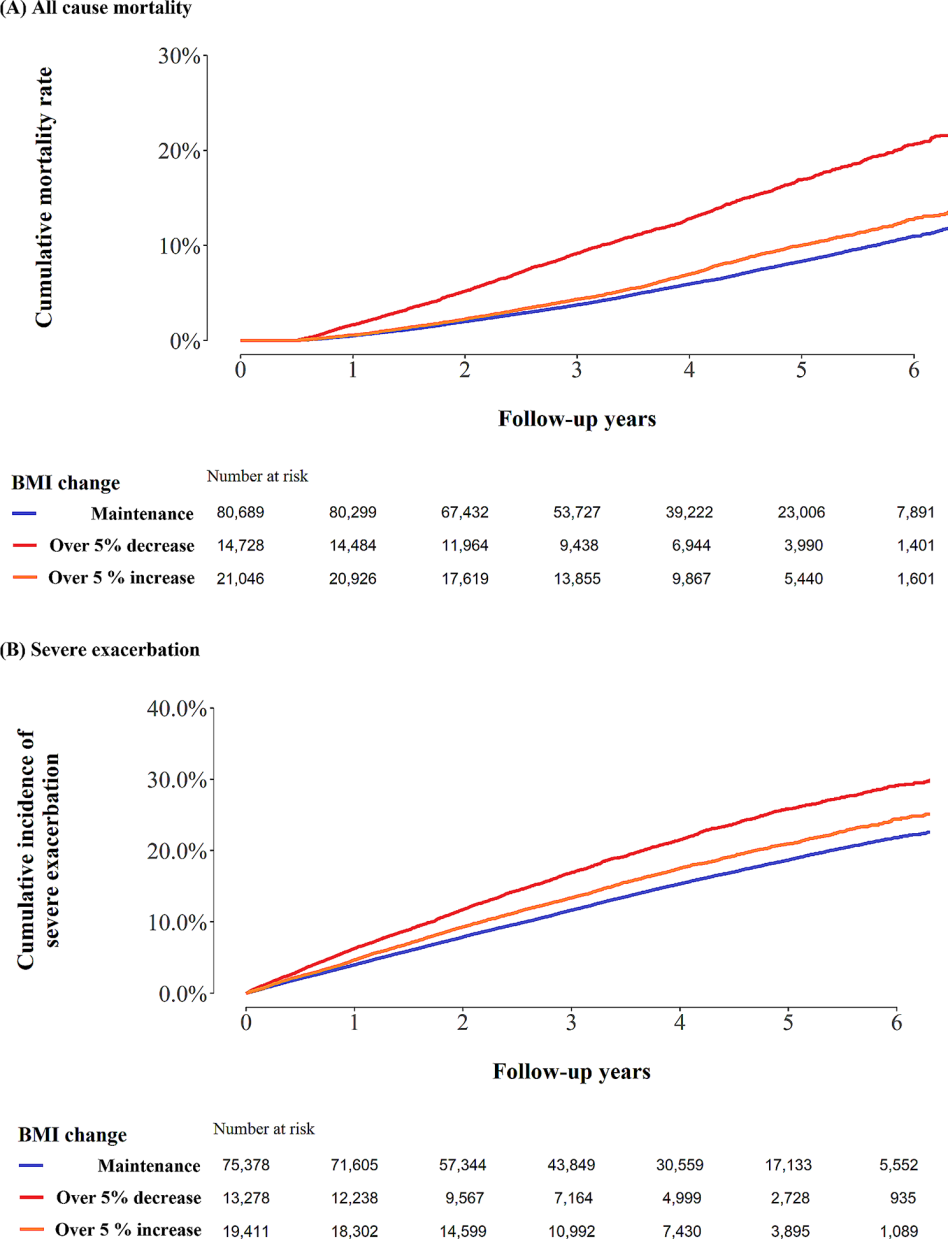
Kaplan Meier curve of (**A**) all-cause mortality and (**B**) severe exacerbation

During a median follow-up of 3.9 years (IQR: 2.5–5.1) from the index date, 8,412 participants died. The mortality rate per 100 person-years was 1.6 in the maintenance group, 3.5 in the decrease group, and 1.9 in the increase group (log-rank test p-values < 0.01, Fig. 1). The adjusted HR for all-cause mortality was 1.70 in the BMI decrease group (95% CI:1.61, 1.79) and 1.13 in the BMI increase group (95% CI:1.07, 1.20), compared to the maintenance group (Table 2).

**Table 2:** Association of BMI change after COPD diagnosis for all-cause mortality and severe exacerbation

	Number of incidence (rate per 100 person-year)	Adjusted^a)^ HR (95% CI)
**All-cause mortality** (***N* = 116,463**)		
Over 5% decrease	1,934 (3.5)	1.70 (1.61, 1.79)
Maintenance	4,981 (1.6)	*Reference*
Over 5% increase	1,497 (1.9)	1.13 (1.07, 1.20)
**Severe exacerbation** (***N* = 108,067**)^b)^		
Over 5% decrease	2,646 (6.0)	1.30 (1.24, 1.35)
Maintenance	10,804 (4.1)	*Reference*
Over 5% increase	3,115 (4.8)	1.12 (1.07, 1.16)

^a)^ Adjusted for age, sex, smoking status, drinking status, residential area, income, CCI, regular MVPA, previous severe exacerbation within a year before Exam 2 (baseline), medication use (ICS, LABA, or LAMA) within 1 year before Exam 2 (baseline), pulmonary TB, bronchiectasis, and pneumonia

^b)^ Patients without a history of previous severe exacerbation between COPD diagnosis and Exam 2 (baseline) were included

BMI, body mass index; COPD, chronic obstructive pulmonary disease; CCI, Charlson Comorbidity Index; CI, confidence interval; HR, hazard ratio; MVPA, moderate-to-vigorous physical activity; ICS, inhaled corticosteroids; LABA, long-acting beta-2 agonist; LAMA, long-acting muscarinic agonist; TB, tuberculosis

**Fig. 2:**
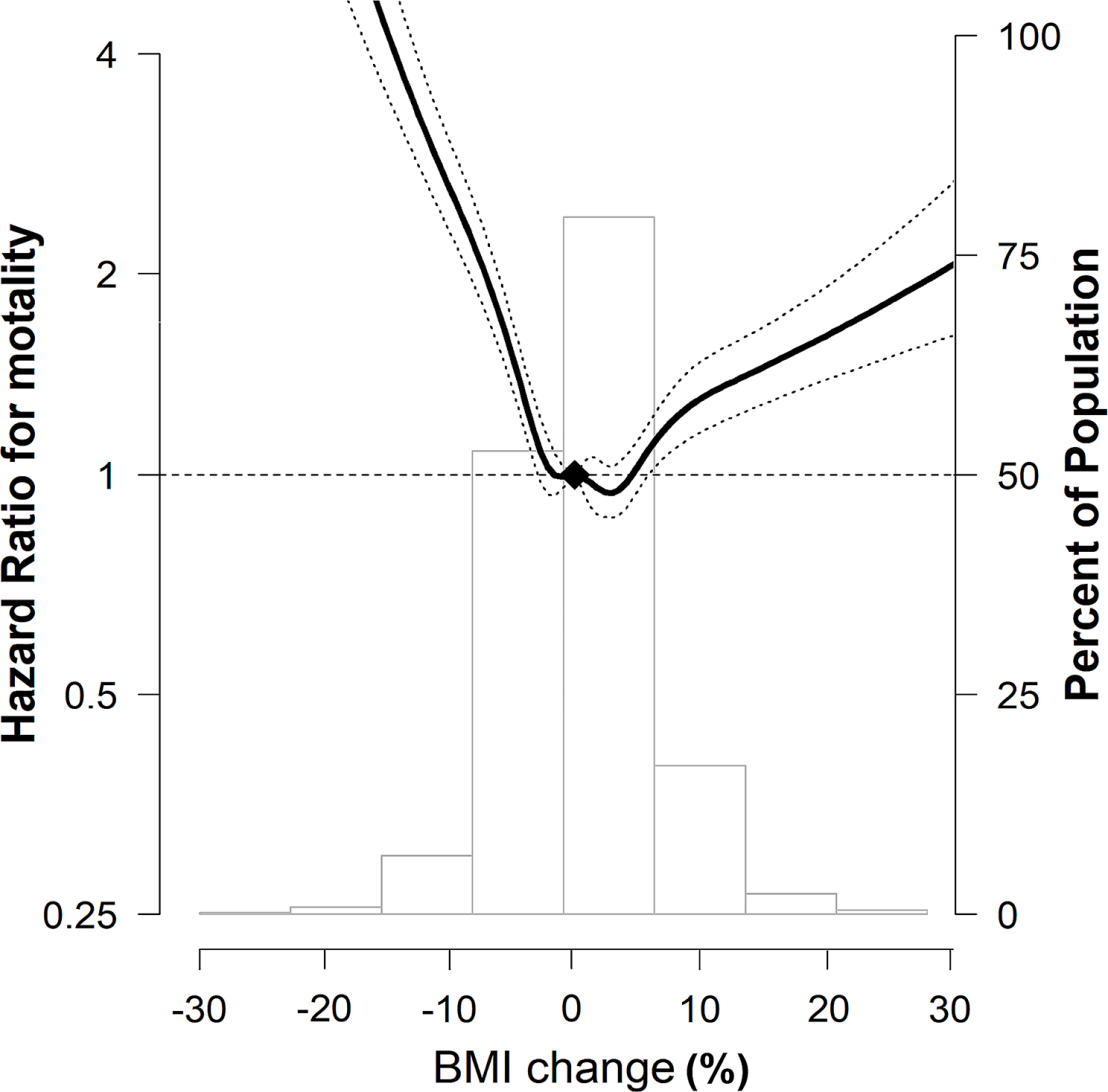
Multivariable-adjusted HRs (95% CI) for all-cause mortality by percentage BMI change The curves represent multivariate-adjusted HRs (solid line) and 95% CIs (dashed lines) for mortality based on restricted cubic splines for percentage BMI change with knots at the 5th, 35th, 65th, and 95th percentiles of sample distribution. The reference value (diamond dot) was set as no change in BMI. The model was adjusted for age, sex, smoking status, drinking status, residential area, income, CCI, regular MVPA, previous severe exacerbation within a year before Exam 2 (baseline), medication use (ICS, LABA, or LAMA) within 1 year before Exam 2 (baseline), pulmonary TB, bronchiectasis, and pneumonia. HR, hazard ratio; CI, confidence interval; BMI, body mass index; CCI, Charlson Comorbidity Index; MVPA, moderate-to-vigorous physical activity; ICS, inhaled corticosteroids; LABA, long-acting beta-2 agonist; LAMA, long-acting muscarinic agonist; TB, tuberculosis

In the spline regression models, the association between percentage BMI change and mortality was approximately nonlinear, indicating that both a decrease and an increase in BMI were associated with an increase in mortality (Fig. 2).

**Fig. 3:**
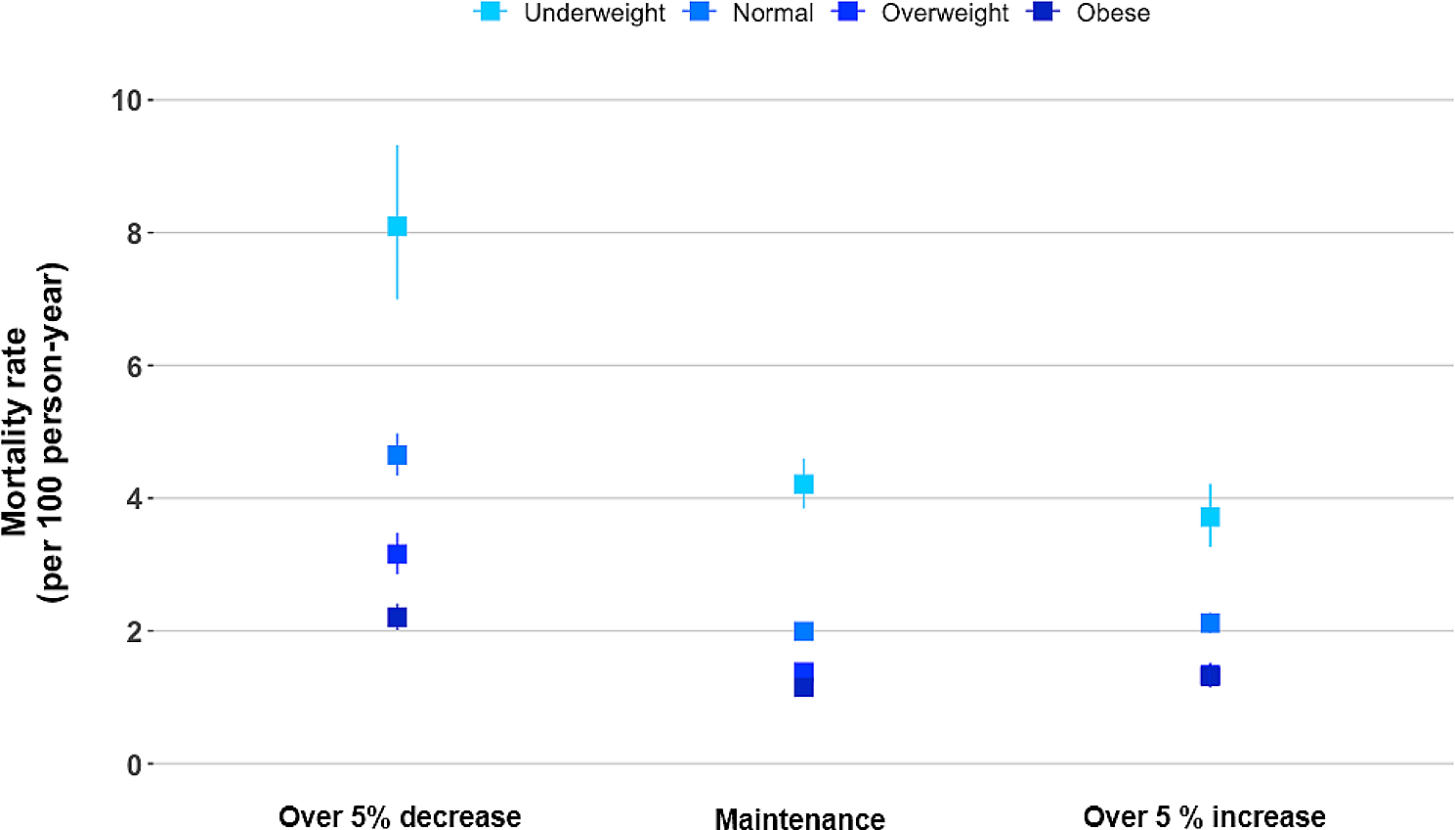
All-cause mortality rate by percentage BMI change according to the BMI before COPD diagnosis. The model was adjusted for age, sex, smoking status, drinking status, residential area, income, CCI, regular MVPA, previous severe exacerbation within a year before Exam 2 (baseline), medication use (ICS, LABA, or LAMA) within 1 year before Exam 2 (baseline), pulmonary TB, bronchiectasis, and pneumonia. BMI, body mass index; COPD, chronic obstructive pulmonary; CCI, Charlson comorbidity index; CI, Confidence Interval; HR, Hazard Ratio; MVPA, moderate-to-vigorous physical activity; ICS, inhaled corticosteroids; LABA, long-acting beta-2 agonist; LAMA, long-acting muscarinic agonist; TB, tuberculosis

In the subgroup analysis according to BMI categories before COPD diagnosis, when they maintained their BMI after COPD diagnosis, the mortality rates per 100 person-years were 4.2, 2.0, 1.4, and 1.1 for underweight, normal weight, overweight, and obese individuals, respectively. When the BMI decreased, the mortality increased regardless of the BMI before COPD diagnosis, with rates per 100 person-years of 8.1, 4.6, 3.2, and 2.2 for underweight, normal weight, overweight, and obese individuals before COPD diagnosis, respectively. When the BMI increased, the mortality rates per 100 person-years were 3.7, 2.1, 1.3 and 1.3 for underweight, normal weight, overweight, and obese individuals before COPD diagnosis, respectively. Patients who were obese before COPD diagnosis only exhibited a significant effect of BMI increase on elevated mortality (Fig. 3; Table 3). This observed relationship was similar in a sensitivity analysis based on the WHO classification of BMI (Supplementary Table 2).

**Table 3:** Association of BMI change after COPD diagnosis for all-cause mortality according to BMI before COPD diagnosis

	Over 5% decrease Adjusted^a)^ HR (95% CI)	Maintenance	Over 5% increase Adjusted^a)^ HR (95% CI)	p for interaction
**BMI before COPD diagnosis**				0.05
Underweight (*N* = 5,545)	1.77 (1.50, 2.09)	*Reference*	0.87 (0.75, 1.02)	
Normal (*N* = 41,286)	1.73 (1.60, 1.88)	*Reference*	1.03 (0.95, 1.12)	
Overweight (*N* = 28,470)	1.75 (1.56, 1.97)	*Reference*	0.98 (0.85, 1.13)	
Obese (*N* = 41,162)	1.62 (1.45, 1.80)	*Reference*	1.18 (1.03, 1.36)	

^a)^ Adjusted for age, sex, smoking status, drinking status, residential area, income, CCI, regular MVPA, previous severe exacerbation within a year before Exam 2 (baseline), medication use (ICS, LABA, or LAMA) within 1 year before Exam 2 (baseline), pulmonary TB, bronchiectasis, and pneumonia

BMI, body mass index; COPD, chronic obstructive pulmonary disease; CCI, Charlson Comorbidity Index; CI, confidence interval; HR, hazard ratio; MVPA, moderate-to-vigorous physical activity; ICS, inhaled corticosteroids; LABA, long-acting beta-2 agonist; LAMA, long-acting muscarinic agonist; TB, tuberculosis

Among severe exacerbation-naïve patients (*N* = 108,067), 16,565 experienced severe exacerbations. The incidence of severe exacerbation per 100-person years was 4.1 in the maintenance group, 6.0 in the decrease group, and 4.8 in the increase group (log-rank test, *p* < 0.01; Fig. 1). The fully adjusted HRs for the risk of severe exacerbation (over 5% BMI decrease vs. maintenance, and over 5% BMI increase vs. maintenance) were 1.30 (95% CI:1.24, 1.35) and 1.12 (95% CI:1.07, 1.16), respectively (Table 2). This result was similar in sensitivity analysis in all patients (*N* = 116,463) adjusting for variables including previous severe exacerbation (Supplementary Table 3).

## Discussion

In this large national cohort study from Korea, a decrease in BMI was associated with an increased risk of severe exacerbation and all-cause mortality in COPD patients. In particular, there was a dose-dependent relationship between a decrease in BMI and all-cause mortality, which was prominent in underweight patients with COPD. In addition, an increase in BMI correlated with an increased risk of death only among obese patients with COPD. Our results highlight that monitoring BMI is important for the non-pharmacological management of COPD and the prediction of outcomes, especially in COPD patients with a low BMI.

Our study extends previous data on the U-shaped association between baseline BMI and clinical outcomes by employing longitudinal changes in BMI in patients with COPD. In particular, the impact on mortality and severe exacerbation were greater when patients with COPD experienced a decrease in BMI than they experienced an increase in BMI (reverse J-shaped curve), and the negative impact of a decrease in BMI on all-cause mortality was more intense in underweight patients with COPD. The observed linear association between a decrease in BMI and increased mortality in our study is consistent with the findings of previous studies [8, 10, 11, 26]. In a similar context, history of previous severe exacerbation, co-existing pulmonary diseases, and comorbidities were more prevalent in the BMI decrease group than in the maintenance and increase groups in our study. Severe exacerbations lead to increased inflammation, metabolic stress, and accelerated muscle wasting [27] and co-existing pulmonary diseases can further exacerbate respiratory symptoms and contribute to a decrease in BMI. Nevertheless, as a decrease in BMI was independently associated with increased mortality, even after adjustment for these covariates, it is important to note that any decrease in BMI in patients with COPD should be closely monitored.

Notably, we showed that a decrease in BMI was associated with a higher risk of all-cause mortality, even in overweight and obese patients with COPD. Epidemiological evidence in patients with cancer has shown that pre-obesity or early obesity status is associated with better outcomes, typically mortality [28]. This phenomenon has been consistently observed in patients with COPD. A meta-regression analysis of five randomized clinical trials revealed that high BMI has a protective effect against lung function decline. The lung function decline was lowest in COPD patients with BMI ≥ 30 kg/m^2^ [4]. Another meta-analysis of 21,150 COPD patients reported that being overweight (25.0–29.9 kg/m^2^) and obese (≥ 30 kg/m^2^) were associated with lower mortality even compared with normal BMI (18.5–24.9 kg/m^2^) [2]. In this way, so-called “obesity paradox” could explain our findings, where overweight or obese status were related to lower risk of death.

An increase in BMI was negatively associated with survival only among obese patients with COPD. Consistent with previous reports, our results suggest that worsening obesity can be detrimental. For example, in a large multinational cohort with moderate COPD, all-cause mortality proportionally increased as BMI increases from 25–<30 kg/m^2^ to ≥ 40 kg/m^2^ [6]. Moderate or severe exacerbations were also higher in obese patients than in patients with normal BMI [6]. The negative impact of BMI increase was remarkable in COPD patients with a predicted forced expiratory volume in 1 s < 50% [8]. Increased cardiovascular and respiratory mortality in obese patients could contribute to an increased risk of death in obese patients with COPD [6]. This result is inconsistent with the findings of a cohort study in Korea [10]. However, in that study, only 16 of 270 patients with COPD experienced an increase in BMI and baseline BMI was lower in the BMI increase group (22.6 kg/m^2^) than in the no change group (23.3 kg/m^2^). These factors could contribute to a lack of persuasiveness owing to the small sample size and different baseline characteristics between the groups.

In addition, we found a 13% reduction in all-cause mortality when underweight COPD patients increased their BMI, although statistical significance was not reached. A few explanations exist. First, the analysis was limited by a relatively small number of underweight patients, which may not enough to draw sufficient statistical power. Previous studies revealed that an increase in BMI, body weight, and body composition was not related to improved survival in underweight patients with COPD [8, 10, 11, 26]. Our results, indicating a trend toward a reduced mortality, may offer a glimpse of evidence suggesting that an increase in BMI in underweight COPD patients could have protective effect on overall survival. Second, a 5% increase in BMI might not be substantial enough to yield a meaningful reduction in mortality. In a large population-based California cohort, weight gain of 5.1–15% showed a relative death risk of 1.09 (95% CI: 0.95, 1.26), while weight gain exceeding 15% demonstrated a significant risk reduction of 10% (RR 0.90, 95% CI: 0.83, 0.98) in underweight individuals [29].

Although this large, nationally representative cohort robustly and comprehensively evaluated the impact of BMI changes on mortality and exacerbation in patients with COPD, several limitations exist. First, as lung function data are not available in the KNHIS database, the definition of COPD was based on the claims data and diagnostic codes, which could lead to misclassification bias. However, this definition has been widely used and validated in several studies [14–16]. Second, there might be potential confounders that were not fully covered in the analysis, including the severity of airflow limitation, which is known to be associated with the risk of mortality in COPD patients [30]. However, severe exacerbations in the previous year, one of predictive factors of mortality in COPD patients [31], were adjusted for mortality. In addition, considering that the obesity paradox is more apparent in COPD patients with severe airway obstruction [5, 8], the generalizability of our results to all patients with COPD should be explored further. Third, the reasons for changes in BMI are unknown. In particular, as it is not known whether outcomes differ based on intentional weight loss in overweight or obese individuals, further studies are necessary. Finally, the K-NHIS lacks data on body composition analyses. One previous study analyzed changes in body composition, such as fat-free mass or fat mass [26]; however, in real clinical practice, assessing changes in BMI would be more realistic and feasible.

## Conclusion

Using a large national cohort study of COPD patients, our research showed a prominent relationship between a decrease in BMI and an increase in all-cause mortality, as well as severe exacerbation. This association was most significant in underweight patients with COPD. Additionally, an increase in BMI increases the risk of death, only among obese patients with COPD. Our study underscores the importance of regularly monitoring BMI changes in patients with COPD.

### Electronic supplementary material

Below is the link to the electronic supplementary material.

Supplementary Material 1

## Data Availability

The data are available from the Korean National Health Insurance Sharing Service (NHISS; https://nhiss.nhis.or.kr/) database, which is open to researchers on request with approval by the Institutional Review Board.
