# Supplementary material for: Longitudinal BMI change and outcomes in Chronic Obstructive Pulmonary Disease: a nationwide population-based cohort study
Source: Respir Res. 2024 Mar 30;25:150. doi: 10.1186/s12931-024-02788-0 (PMC10981805; doi:10.1186/s12931-024-02788-0)
Supplement: Supplementary file 1 — Supplementary Material 1 [file 12931_2024_2788_MOESM1_ESM.docx]

SUPPLEMENTARY MATERIALS

**Longitudinal BMI change and Outcomes in Chronic Obstructive Pulmonary Disease: A Nationwide Population-based Cohort Study**

Taeyun Kim, MD^1*^, Sun Hye Shin, MD, PhD^1*^, Hyunsoo Kim, MS^2^*, Yunjoo Im, MD, PhD^1^, Juhee Cho, PhD^2,3^, Danbee Kang, PhD^2,3¶^, Hye Yun Park, MD, PhD^1¶^

^1^Division of Pulmonary and Critical Care Medicine, Department of Internal Medicine, Samsung Medical Center, Sungkyunkwan University School of Medicine, Seoul, Republic of Korea

^2^Center for Clinical Epidemiology, Samsung Medical Center, Seoul, Republic of Korea

^3^Department of Clinical Research Design and Evaluation, SAIHST, Sungkyunkwan University, Seoul, South Korea

**A Table of Contents**

**Supplementary Tables**

**Supplementary Table 1.** Numbers (percentages) of individuals changed BMI from baseline.

**Supplementary Table 2.** Association of BMI change after COPD diagnosis for all-cause mortality according to WHO BMI classification before COPD diagnosis

**Supplementary Table 3.** Association of BMI change after COPD diagnosis for severe exacerbation (N = 116,463)

**Supplementary Table 1.** Numbers (percentages) of individuals changed BMI from baseline.

1. Asian-specific BMI classification

|  | Over 5% decrease | Maintenance | Over 5% increase |
| --- | --- | --- | --- |
| **BMI before COPD diagnosis** |  |  |  |
| Underweight (N =5,545) | 677 (12.2) | 3,122 (56.3) | 1,746 (31.5) |
| Normal (N =41,286) | 4,853 (11.7) | 26,868 (65.1) | 9,565 (23.2) |
| Overweight (N =28,470) | 3,379 (11.9) | 20,425 (71.7) | 4,666 (16.4) |
| Obese (N = 41,162) | 5,819 (14.1) | 30,274 (73.6) | 5,069 (12.3) |

1. WHO BMI classification

|  | Over 5% decrease | Maintenance | Over 5% increase |
| --- | --- | --- | --- |
| **BMI before COPD diagnosis** |  |  |  |
| Underweight (N = 5,545) | 677 (12.2) | 3,122 (56.3) | 1,746 (31.5) |
| Normal (N = 69,756) | 8,232 (11.8) | 47,293 (67.8) | 14,231 (20.4) |
| Overweight (N =36,138) | 4,898 (13.6) | 26,747 (74.0) | 4,493 (12.4) |
| Obese (N = 5,024) | 921 (18.3) | 3,527 (70.2) | 576 (11.5) |

BMI, body mass index; COPD, chronic obstructive pulmonary disease; WHO, world health organization.

**Supplementary Table 2.** Association of BMI change after COPD diagnosis for all-cause mortality according to WHO BMI classification before COPD diagnosis

|  | Over 5% decrease Adjusted^a)^ HR (95% CI) | Maintenance | Over 5% increase Adjusted^a)^ HR (95% CI) | p for interaction |
| --- | --- | --- | --- | --- |
| **BMI^b)^ before COPD diagnosis** |  |  |  | 0.05 |
| Underweight (N = 5,545) | **1.77 (1.50, 2.09)** | *Reference* | 0.87 (0.75, 1.02) |  |
| Normal (N = 69,756) | **1.75 (1.64, 1.87)** | *Reference* | 1.05 (0.98, 1.13) |  |
| Overweight (N =36,138) | **1.62 (1.45, 1.81)** | *Reference* | **1.16 (1.01, 1.34)** |  |
| Obese (N = 5,024) | **1.52 (1.11, 2.09)** | *Reference* | 1.33 (0.87, 2.04) |  |

^a)^ Adjusted for age, sex, smoking status, drinking status, residential area, income, CCI, regular MVPA, previous severe exacerbation within a year before Exam 2 (baseline), medication use (ICS, LABA, or LAMA) within 1 year before Exam 2 (baseline), pulmonary TB, bronchiectasis, and pneumonia.

^b)^ BMI was classified based on WHO classification: underweight (< 18.5 kg/m^2^), normal (18.5 – 24.9 kg/m^2^), Overweight (25 – 29.9 kg/m^2^), and Obese (≥ 30 kg/m^2^).

BMI, body mass index; COPD, chronic obstructive pulmonary disease; WHO, world health organization; CCI, Charlson Comorbidity Index; CI, confidence interval; HR, hazard ratio; MVPA, moderate-to-vigorous physical activity; ICS, inhaled corticosteroids; LABA, long-acting beta‐2 agonist; LAMA, long-acting muscarinic agonist; TB, tuberculosis

**Supplementary Table 3**. Association of BMI change after COPD diagnosis for severe exacerbation (N = 116,463)

|  | **Number of incidence**  **(100 person-year)** | **Adjusted^a)^ HR**  **(95% CI)** |
| --- | --- | --- |
| **Severe exacerbation** |  |  |
| Over 5% decrease | 3,593 (7.6) | 1.31 (1.26, 1.37) |
| Maintenance | 13,571 (5.0) | Reference |
| Over 5% increase | 4,048 (5.8) | 1.12 (1.08, 1.16) |

^a)^ Adjusted for age, sex, smoking status, drinking status, residential area, income, CCI, regular MVPA, previous severe exacerbation within a year before Exam 2 (baseline), medication use (ICS, LABA, or LAMA) within 1 year before Exam 2 (baseline), pulmonary TB, bronchiectasis, and pneumonia.

BMI, body mass index; COPD, chronic obstructive pulmonary disease; CCI, Charlson Comorbidity Index; CI, confidence interval; HR, hazard ratio; MVPA, moderate-to-vigorous physical activity; ICS, inhaled corticosteroids; LABA, long-acting beta‐2 agonist; LAMA, long-acting muscarinic agonist; TB, tuberculosis
